# Familial and genetic association with neurodevelopmental disorders caused by a heterozygous variant in the *SRRM2* gene

**DOI:** 10.3389/fendo.2023.1240168

**Published:** 2023-08-09

**Authors:** Tao Zhang, Lei Xu, Hongdan Zhu, Yuyi Ying, Jinlong Ding, Haigang Ding, Xiaoliang Shi, Yao He, Xin Jin, Guiyu Xia

**Affiliations:** ^1^ Shaoxing Maternity and Child Health Care Hospital, Shaoxing, Zhejiang, China; ^2^ Obstetrics and Gynecology Hospital of Shaoxing University, Shaoxing, Zhejiang, China; ^3^ Shaoxing University, Shaoxing, Zhejiang, China

**Keywords:** neurodevelopmental disorders, *SRRM2* gene, whole exome sequencing, 3D structure, prenatal diagnosis

## Abstract

**Background:**

Neurodevelopmental disorders (NDDs) are a class of disorders affecting brain development and function, characterized by an inability to reach cognitive, emotional, and motor developmental milestones. The pathology of NDDs is complex. A recent study found that variants in the *SRRM2* gene cause NDDs. However, genetic conditions play the most important role in the etiology of NDD. The genetic causes of NDD are extremely heterogeneous, leading to certain challenges in clinical diagnosis.

**Methods:**

A pregnant woman with congenital intelligence disorder came to our hospital for genetic diagnosis to predict the status of her fetus. Her mother and a brother also suffer from congenital intelligence disorder. She has a daughter with speech delay. Whole exome sequencing was used to identify a mutation (c.1415C>G) in the *SRRM2 gene* of this family that resulted in a change in the 472nd amino acid residue of the SRRM2 protein from serine to terminated.

**Conclusion:**

We report a family with an autosomal dominant genetic disorder caused by variants in the *SRRM2* gene causing NDDs. Prenatal diagnosis can help patients with this genetic disorder to have healthy offspring.

## Introduction

Neurodevelopmental disorders (NDDs) (OMIM#616,521) are defined as a class of disorders affecting brain development and function, largely determined by genetics, that result in behavioral, cognitive, sensory, and motor changes, as well as speech and language deficits. Due to the genetic heterogeneity of NDDs, it has hindered the clinical identification of molecular etiology in patients. The pathogenesis of NDDs is complex, and the genetic basis of most NDDs patients remains unclear.

It has recently been found 22 NDDs patients with heterozygous loss-of-function variants in *SRRM2* gene and described the phenotype associated with *SRRM2* variants (1). It was also found that SRRM2 deletion leads to cassette exon skipping and weak splice sites in short introns that tends to alter large protein structural domains (2). Abnormal function of SRRM2 has been found to be associated with other human diseases. Reduction of long SRRM2 transcripts and increase of short transcripts were found in peripheral blood and substantia nigra of Parkinson’s patients (3). Phosphorylated nuclear speckle (NS) scaffold protein SRRM2 is mislocalized in Alzheimer’s disease, and the mislocalization of SRRM2 is correlated with the severity of pathological tau deposition (4). The S346F mutation in *SRRM2* tends to papillary thyroid carcinoma by affecting selective splicing of unknown downstream target genes (5). However, there are no other studies on variants in *SRRM2* gene causing NDDs.

RNA splicing, the conversion of pre-messenger RNA to mature mRNA, is a highly conserved process, and this process is performed by the spliceosome. The spliceosome consists of five small nuclear ribonucleoprotein particles (snRNPs: U1, U2, U4, U5, and U6) and other protein factors. Among them, SR proteins are an evolutionarily conserved family characterized by one or two N-terminal RNA recognition motifs and an arginine-serine rich downstream region (RS domain), of which SRm300 encoded by *SRRM2* gene is one of the protein factors. SRRM2 is the core structural component of NS and form a scaffold with SON to bind to the proteins in NS (6). Splicing of precursor mRNA enables diversity of transcriptome and proteome expression in a tissue-specific manner and is a key mechanism for increasing protein complexity in humans (7). Alterations and errors in splicing regulation caused by splicing factors are closely related to many diseases.

In this study, we report a family with NDDs characterized by congenital intellectual disability with functional deficits. Whole exome sequencing revealed a heterozygous nonsense variant (c.1415C>G) in *SRRM2*. The pathogenic variant follows an autosomal dominant pattern of inheritance. This variant results in a change in amino acid position 472 from serine to terminated, leading to the premature termination of SRRM2 protein sequence and the change of protein structure, which may affect the RNA splicing process in which SRRM2 is involved in, thus affecting the development of neurointelligence. Our identification of NDDs caused by *SRRM2* gene variants in a family has important implications for further studies on the clinical diagnosis and pathogenesis of NDDs. We offered prenatal diagnosis service for the pregnant woman at 18 weeks of pregnancy, and the final results show that the fetus carried the wild genotype in *SRRM2* gene.

## Materials and methods

### Sample collection

The study was approved by the institutional ethics committee of Shaoxing Maternity and Child Health Care Hospital, which have signed an informed consent document. Peripheral blood samples were also collected from the patient and her family members. Parental consent was obtained for collecting the prenatal fetal amniotic fluid at 18 weeks of pregnancy.

### Copy number variation sequencing

gDNA of fetus was sheared into 150–200 bp fragments by enzyme digestion reagent. After end repair and addition of an A overhang and adaptor ligation, PCR was used to amplify DNA fragments. Fourteen to 48 PCR amplification products were selected according to the amount of data required. After the mixing, the total amount of 160 ng DNA was taken for single-strand separation and cyclization reaction, and finally a single-strand circular DNA library with a joint was obtained. Finally, BGISEQ-500, an autonomous sequencing platform, was used for single-end sequencing for 35 cycles. The sequencing quality of raw reads was first evaluated to remove low-quality and adaptor contaminated reads. Then, we use the SOAP (8) and compare the GRCh37 (hg19) genome sequence to remove PCR duplicates and select only uniquely mapped reads. The analytic method was performed as described before (9).

### Whole exome sequencing

Genomic DNA (gDNA) was extracted from the patient and her family blood using a DNEasy Blood and Tissue Kit (Qiagen, Hilden, Germany) according to the manufacturer’s procedures, respectively. For WES, the gDNA of the proband was enriched for coding exons using Agilent SureSelect Low Input Reagent Kit and sequenced on Illumina HiSeq X Ten platform. The sequencing data captured 99.75% of coding regions across 35,519,957 bp length of 25,701 genes in total. The average sequence depth is 180.347X and 97.87% of targeted regions with average depth of > 20X.

### Data analysis

We used 1000 Genomes database (1000 human genome dataset), Genome AD (Genome Aggregation Database dataset) 2.1.1, and ExAC (The Exome Aggregation Consortium dataset) to screen the SNV and indels and the OMIM, HGMD, and Clinvar databases to filter the reported mutations. dbNSFP database was used to predict the pathogenicity of missense mutation and splice mutation. All mutation sites were classified by ACMG genetic variation classification criteria and guidelines. Finally, Sanger sequencing method was used to verify all possible pathogenic sites.

### Protein structure prediction

The protein sequence with 2,752 amino acid residues of SRRM2 was downloaded from NCBI. The wild-type and mutant-type 3D structure of the SRRM2 protein was predicted using SWISS-MODEL web server (https://swissmodel.expasy.org/) (10) and UniProt database (https://alphafold.ebi.ac.uk/). The best model was selected based on QMEANDisCo global score. The final predicted structure was visualized using PyMOL program (https://pymol.org/).

## Results

### Clinical features

**Figure 1:**
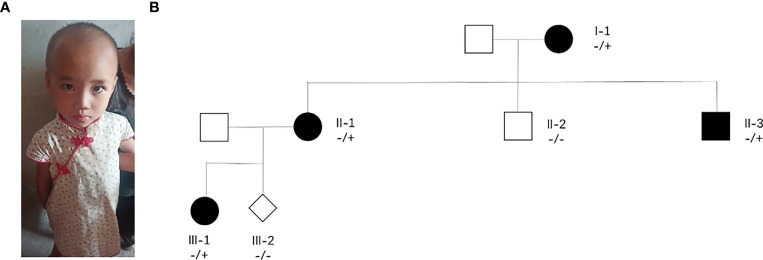
Major clinical phenotype and family tree. **(A)** Image of the pregnant woman’s daughter who have a speech delay. **(B)** II-1 are pregnant women with neurodevelopmental disorders.

A pregnant woman with congenital intelligence disorder, speech delay, and special facial features came to our hospital for prenatal diagnosis. She had previously given birth to a daughter with delayed speech and unusual facial features (**Figure 1A**). In this pregnant woman’s family, her mother and a brother also had congenital intellectual disorders and communication barriers, whereas her father and another brother were phenotypically normal, and her husband was a phenotypically normal individual, without any family history of genetic diseases (**Figure 1B**).

### Identification of variant in *SRRM2* gene

**Figure 2:**
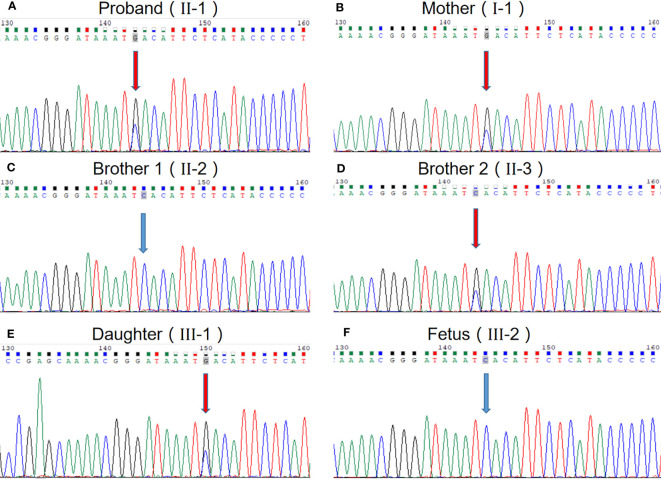
The evidence of the heterozygous nonsense mutation c.1415C> G in the *SRRM2* gene. **(A)** The proband; **(B)** mother of the proband; **(C)** one brother of the proband; **(D)** another brother of the proband; **(E)** daughter of the proband; **(F)** fetus of the proband.

We performed WES to detect presence of any mutation(s) in the related disease-causing genes. The sequencing was performed using capture high-throughput chip technology, detection of almost 20,000 genes in the human genome. Sanger sequence was used to verify the mutations. The results showed that a suspected disease-causing variant, uc002crk.3; c.1415C>G; p.Ser472* in *SRRM2* gene located on chromosome Chr16, which is a heterozygous gene mutation of hereditary NDD in this family. The heterozygous mutation, a nonsense mutation caused by the substitution of nucleotide C with G at 1415 of the gene coding sequence, resulting in the serine codon at position 472 being changed to a stop codon. We subsequently collected blood samples from the patient and the patient’s mother, two brothers, and daughter and verified the mutation by sanger sequencing, which confirmed that the variant (uc002crk.3; c.1415C>G; p.Ser472*) in *SRRM2* gene is inherited from the mother and is the major cause of NDD in this family (**Figure 2**). The sequence data had been submitted to GenBank with the accession number 2712395. Prenatal diagnosis results show that the fetus carried the wild genotype in *SRRM2* gene.

### Effect of this variant on protein structure

**Figure 3:**
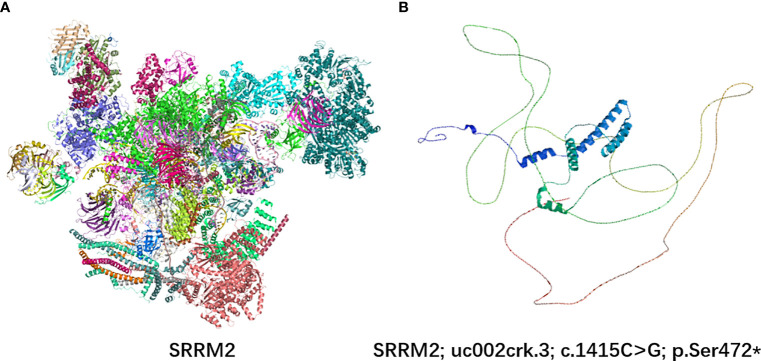
3D protein simulation of SRRM2 in UniProt and SWISS-MODEL web server. **(A)** The wild-type structure of SRRM2; **(B)** The mutant prtein structure predicted by SWISS-MODEL.

SRRM2 contains a serine-arginine-enriched RS domain that appears to facilitate interactions between mRNA and the spliceosome catalytic machinery, participating in splicing regulation by facilitating protein and protein/RNA interactions. In order to analyze this variant on protein structure, we used SWISS-MODEL web server (10) to predict changes in SRRM2 protein structures when the *SRRM2* sequence was mutated (**Figure 3**). The premature appearance of the stop codon leads to significant changes in the structure of SRRM2 protein, which may affect its function in RNA splicing.

## Discussion

SRRM2 has recently been reported as an important gene in NDDs. Cuinat et al. report 22 cases of NDDs caused by mutations in SRRM2, of which 12 had shifted variants, eight had nonsense variants, and two had microdeletions of 66 and 270 kb (1). These mutations were all heterozygous mutations. The 22 patients had developmental delays, mainly speech delay, and four of the 20 patients did not have intellectual disability. Moderate or severe intellectual disability was observed in these patients. Some motor deficits were present in these patients. The weight of these patients was generally high. Ophthalmic and visceral abnormalities were present in some patients (1).

Active chromatin regions are associated with NSs involved in RNA processing. SRRM2 and SON are important components that make up the NS. SRMM2 and SON form the scaffold that binds the proteins in NS together. When SON is knocked down, it leads to partial dissociation of SRRM2, RBM25, PNN, and SRRM1, which are localized to the NS, from the NS. But this dissociation is lower when SRRM2 is knocked down relative to the former. In the presence of both intrinsically disordered regions of SRRM2 and knockdown of SON, the NS structure is almost completely depolymerized (6).

Disruption of NSs by knockdown of *srrm2* leads to an overall reduction of chromatin interactions in the active region (11). SRRM2 deletion causes cassette exons with short introns and weak splice sites to be skipped, tending to alter large protein structural domains (2). The deletion of SRRM2 disrupts the NSs and alters gene expression. Disruption of NSs reduces chromatin interactions in active compartments (11). *SRRM2* missense mutations may affect splicing of downstream genes and lead to susceptibility to PTC (5).

**Figure 4:**
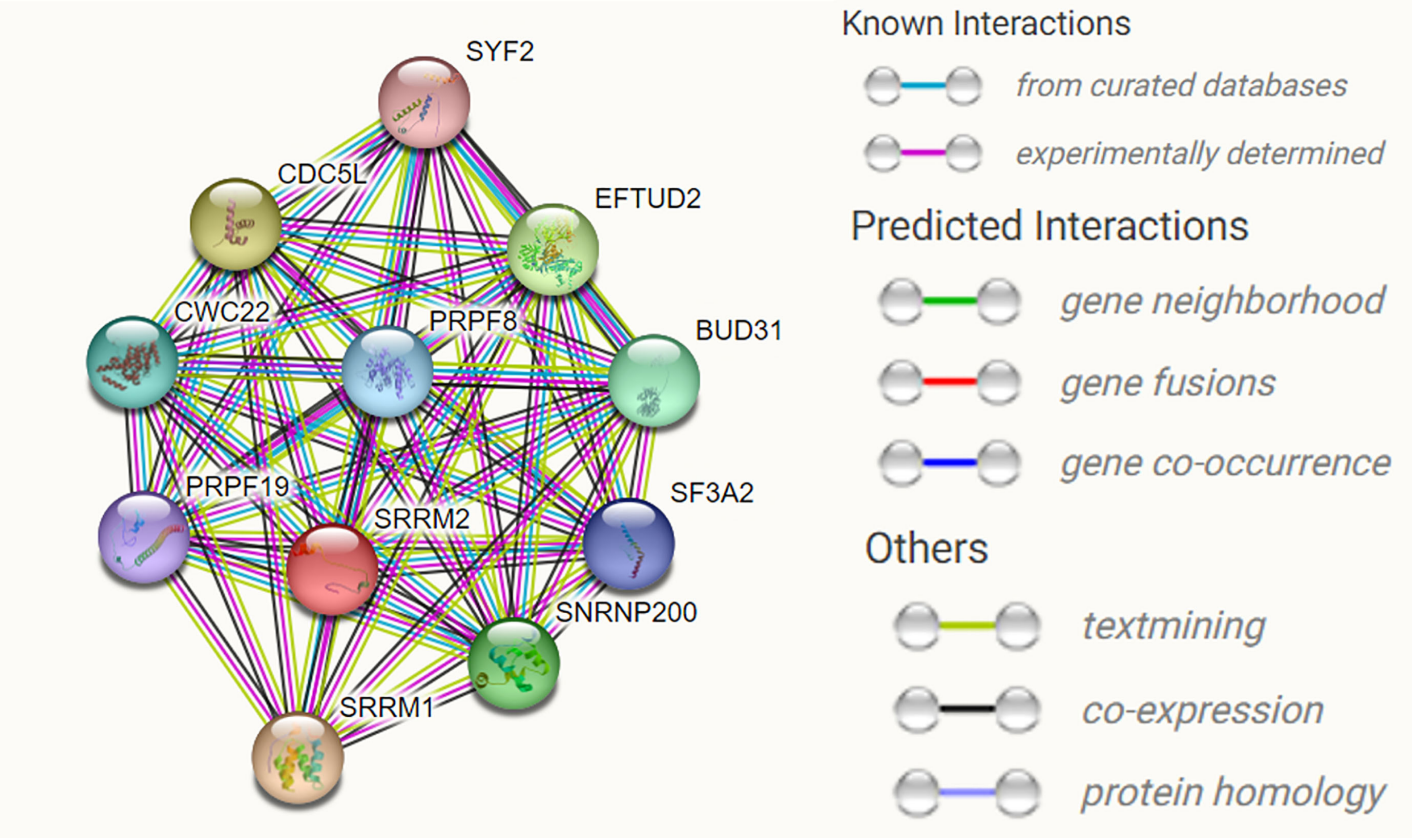
The predicted interacting proteins of SRRM2 using STRING website. Multiple proteins involved in RNA splicing, namely, SRRM1,EFTUD2, SNRNP200, BUD31, CWC22, PRPF8, SF3A2, PRPF19, and SYF2, as well as transcription factor CDC5L.

SRRM2 encodes the SRm300 protein, which contains a structural domain rich in alternating serine and arginine residues but lacks an RNA recognition motif. SRm300 contains a highly conserved N-terminal structural domain consisting of several unique repeat motifs rich in serine, arginine and proline residues, and two very long polyserine bundles (12). SRm160 and SRm300 form a complex required for specific pre-mRNA splicing. Binding of SRm160/300 to pre-mRNA is normally dependent on U1 snRNP and is stabilized by U2 snRNP (13). SRm300, an SR-related protein at the core of the human catalytic spliceosome, is a functional direct homolog of yeast Cwc21p. SRm300 interacts directly with two key splicing factors binding Prp8p and Snu114p (14). The role of Cwc21p in localizing the 3’ splice site during the transition of the spliceosome to the second step of conformation is mediated through its interaction with U5 snRNP (15). We used STING software to predict the interacting proteins with SRRM2 and found multiple proteins involved in RNA splicing, namely, SRRM1, EFTUD2, SNRNP200, BUD31, CWC22, PRPF8, SF3A2, PRPF19, and SYF2, as well as transcription factor CDC5L (**Figure 4**) involved in transcription regulation (**Table 1**).

**Table 1:** Names and functions of predicted SRRM2 interacting proteins.

Interacting proteins	Function of proteins
**SRRM1**	Serine/arginine repetitive matrix protein 1; part of pre- and post-splicing multiprotein mRNP complexes. Involved in numerous pre-mRNA processing events.
**CDC5L**	Cell division cycle 5–like protein; DNA-binding protein involved in cell cycle control. May act as a transcription activator.
**EFTUD2**	116 kDa U5 small nuclear ribonucleoprotein component; component of the U5 snRNP and the U4/U6-U5 tri-snRNP complex required for pre-mRNA splicing.
**SNRNP200**	U5 small nuclear ribonucleoprotein 200 kDa helicase; RNA helicase that plays an essential role in pre-mRNA splicing as component of the U5 snRNP and U4/U6-U5 tri-snRNP complexes.
**BUD31**	Bud site selection protein 31; Protein BUD31 homolog; Spliceosomal Bact complex.
**CWC22**	Pre-mRNA-splicing factor CWC22 homolog; Required for pre-mRNA splicing and for exon-junction complex (EJC) assembly.
**PRPF8**	Pre-mRNA-processing-splicing factor 8; functions as a scaffold that mediates the ordered assembly of spliceosomal proteins and snRNAs.
**SF3A2**	Splicing factor 3A subunit 2; subunit of the splicing factor SF3A required for “A” complex assembly formed by the stable binding of U2 snRNP to the branchpoint sequence (BPS) in pre-mRNA.
**PRPF19**	Pre-mRNA-processing factor 19; ubiquitin-protein ligase, which is a core component of several complexes mainly involved pre-mRNA splicing and DNA repair.
**SYF2**	Pre-mRNA-splicing factor SYF2; may be involved in pre-mRNA splicing; belongs to the SYF2 family.

In this case, we report a neurodevelopmental disorder in a line that is caused by a nonsense mutation in *SRRM2* (uc002crk.3; c.1415C>G; p.Ser472*). The mutation is also a heterozygous mutation. Patients in this pedigree exhibit intellectually disabled, delayed language development, and dysmorphic facial features. The heterozygous nonsense mutation in proband was inherited from her mother. A case with a more severe functional deletion of *SRRM2* (c.838C>T) was identified in the decipher genomics database and was found to present a similar clinical phenotype to the patient in this case.

In summary, by whole exome sequencing, we found that the causative variant (uc002crk.3; c.1415C>G; p.Ser472*) in *SRRM2* gene for NDDs. This is the first report of neurodevelopmental disorder caused by *SRRM2* gene variant in a family. This variant is reported for the first time, which further improves the database of genetic pathogenic variants in NDDs.

## Data availability statement

The datasets presented in this study can be found in online repositories. The names of the repository/repositories and accession number(s) can be found in the article/supplementary material.

## Ethics statement

The studies involving human participants were reviewed and approved by Institutional ethics committee of Shaoxing Maternity and Child Health Care Hospital. Written informed consent to participate in this study was provided by the participants’ legal guardian/next of kin. Written informed consent was obtained from the individual(s), and minor(s)’ legal guardian/next of kin, for the publication of any potentially identifiable images or data included in this article.

## Author contributions

TZ and HZ conceived and designed the experiments. LX and YY performed the experiments, XS, JD, and HD contributed new materials. YH, XJ, and GX analyzed the data and wrote the paper. All authors read and improved the manuscript. All authors contributed to the article and approved the submitted version.
